# Povidone-iodine pleurodesis versus talc pleurodesis in preventing recurrence of malignant pleural effusion

**DOI:** 10.1186/s13019-015-0270-5

**Published:** 2015-05-01

**Authors:** Islam M Ibrahim, Ahmed L Dokhan, Alaa A El-Sessy, Mohammed F Eltaweel

**Affiliations:** 1 Cardiothoracic surgery department, Faculty of Medicine, Menoufia University, Yassin Abd El-Ghaffar Street, 32511 Shepin El-Kom, Menoufia, Egypt; 2 General surgery department, Faculty of Medicine, Menoufia University, Menoufia, Egypt

**Keywords:** Pleural effusion, Pleural space (drainage, Management), Pleurodesis

## Abstract

**Background:**

Malignant pleural effusions continue to be a common problem in patients with metastatic disease, leading to a significant reduction in quality of life with progressive dyspnea, dry cough, chest pain and reduced physical activity. This study was conducted to compare the efficacy, safety, and outcome of Talc Powder Pleurodesis (TPP) with Povidone-iodine Pleurodesis (PIP) through a chest drain as a palliative preventive treatment of recurrent malignant pleural effusion.

**Methods:**

A total of 39 neoplastic patients with recurrent malignant pleural effusion were enrolled in a prospective randomized trial. Twenty-one patients received Talc pleurodesis (group A), and eighteen patients (group B) received Povidone-iodine pleurodesis. The continuous variables were expressed as mean values ± standard deviation (SD) and compared using the unpaired t-test. The discrete variables were expressed as percentage and compared using the chi-square test (χ^2^) test. *p*-values of less than 0.05 were considered significant.

**Results:**

Our study included 11 males and 28 females, the mean age was (71.0 ± 5.0) years for group A and (70.9 ± 5.1) years for group B (non-significant). Post-procedure analgesic requirements were recorded in both groups. Four patients in each group had fever (>38°C) within 48 hours of the procedure. Both groups achieved good symptomatic relief. There were no in-hospital deaths. The mean post-procedure hospital stay was (4.7 ± 1.2) days for group A and (4.2 ± 1.0) for group B (non-significant). At follow-up recurrence of significant pleural effusion requiring intervention was noted in four and five patients in group A and group B, respectively (non-significant difference).

**Conclusion:**

Povidone-iodine pleurodesis can be considered as a good alternative to Talc pleurodesis for recurrent malignant pleural effusion. The drug is available, cost effective, safe and can be administered through an intercostal drain and repeated if necessary.

## Background

Pleural effusion is the accumulation of fluid in the pleural space caused by many conditions, the commonest of which are; congestive heart failure, pneumonia and malignancy [1]. Malignant pleural effusions continue to be a common problem in patients with metastatic disease, leading to a significant reduction in quality of life with progressive dyspnea, dry cough, chest pain and reduced physical activity [2]. The commonest cause of malignant pleural effusion is bronchogenic carcinoma followed by metastatic breast cancer [3]. The management of recurrent malignant pleural effusions is palliative, and should be aiming at improving the quality of life with minimal complications. The aim of pleurodesis in these patients is to prevent re-accumulation of the effusion and thereby of symptoms, and avoid the high cost and physical and emotional trauma caused by repeated hospitalization for thoracocentesis [4].

Over the past several years, chemical pleurodesis has evolved as the most widely accepted treatment method for these conditions [5]. There are a wide variety of agents available for pleurodesis, such as tetracycline derivatives (doxycycline or minocycline), talc (insufflation or slurry), bleomycin, mitoxantrone, nitrogen mustard, silver nitrate, iodopovidone, dry killed Corynebacterium parvum and OK-432 (obtained from the Su strain of Streptococcus pyogenes) [6]. Like any other drug, the criteria for selection of the agent for pleurodesis include its effectiveness, affordability, availability, ease of administration and safety profile [7].

Many reports showed talc pleurodesis as the surgical pleurodesis of choice for recurrent malignant effusion, with a reported success rate of 90% [8,9]. In Egypt, however, the use of talc powder has been disapproved and it remains unavailable in the Egyptian market [4]. Instead, bleomycin, which is expensive and less effective, is being used.

Povidone–iodine (in a 10% solution), which is primarily used as a topical antiseptic agent, has recently been shown to be an inexpensive, easily available, safe, and mostly effective alternative sclerosing agent in some series [5]. It also can be infused, with excellent tolerance, through intercostal drain under local anesthesia and repeated, if necessary [4].

This study was conducted to compare the efficacy, safety, and outcome of Talc Powder Pleurodesis (TPP) with Povidone-iodine Pleurodesis (PIP) through a chest drain as a palliative preventive treatment of recurrent malignant pleural effusion.

## Methods

This study was conducted at the cardiothoracic Surgery department in Menoufia University Hospitals between January and November 2013. A total of 39 patients with malignant pleural effusion were enrolled in a randomized non-blinded controlled trial. Informed consent was obtained from each patient and ethical approval for the study was obtained from the local ethics committee. All patients diagnosed (clinically and histo-pathologically) with recurrent malignant pleural effusion were included in our study. Patients with allergy to iodine and those with incompletely inflated lung on radiograph were excluded from the study.

Therapeutic thoracocentesis was performed in all patients, and the drained pleural fluid amounts were recorded and sent for physical, biochemical, bacteriological and cytological evaluation. Patients were then randomized (using simple randomization with allocation concealment) into two groups; group A (21 patients), and group B (18 patients).

### Technique of pleurodesis

After insertion of wide-pore chest drain (size 28 F - 36 F) under local anesthesia and allowing for free drainage of pleural fluid over 6–12 hours, chest radiograph were done to confirm the drainage of fluid and inflation of the lung.

For patients in group A, a dose of 5 grams of sterile, asbestos-free talc (Steritalc® F2, manufactured by Novatech, France) in 50 ml of normal saline were instilled through the chest drain. The chest drain was clamped for 6 hours after talc instillation.

For patients in group B, 20 ml of 10% Povidone-iodine (Betadine®, manufactured by Nile Co. for Pharmaceuticals and Chemical Industries, Cairo, Egypt; licensed by Mundi Pharma AG, Basel, Switzerland) (Povidone-iodine pH: 4.5 – 5.5) mixed with 10 ml of lidocaine 1% and 30 ml of normal saline were instilled through the chest drain, which was clamped for 6 hours as well.

Chest drains were removed when the chest radiograph confirmed satisfactory lung expansion, and the total 24-hour drainage was less than 100 ml, with no air leak. Another chest radiograph was done for all patients few hours post chest drain removal and if satisfactory, patients were discharge on the same day. Pain was assessed and scaled using comparative pain scale into; minor pain (does not interfere with most activities, able to adapt to pain psychologically and with medication or devices such as cushions), moderate pain (interferes with many activities, requires lifestyle changes but patient remains independent, unable to adapt to pain) and severe pain (Unable to engage in normal activities, patient is disabled and unable to function independently). Complications such as fever, allergic Reactions and empyema were recorded.

### Follow-up

All patients were followed-up in the out-patient clinic, after 2 weeks, 2 months and 6 months. The efficacy of pleurodesis was defined in three levels of response: complete (absence of pleural fluid re-accumulation), partial (residual pleural fluid or re-accumulation, which did not require further drainage or remained asymptomatic), and failed (additional pleural procedures were necessary). A normal chest radiograph or radiological re-accumulation of pleural fluid without recurrence of dyspnea or the need for drainage was reported as a success.

### Statistical analysis

The continuous variables were expressed as mean values ± standard deviation (SD) and compared using the unpaired t-test. The discrete variables were expressed as percentage and compared using the chi-square test (χ^2^) test. *p*-values of less than 0.05 were considered significant.

## Results

A total of 39 patients with malignant pleural effusion were enrolled during the study period and randomized into two groups; twenty-one patients in group A, underwent Talc powder pleurodesis, while eighteen patients in group B underwent Povidone-iodine pleurodesis through the intercostal chest drain.

They were 11 males (28.2%) and 28 females (71.8%). Their ages ranged from 65–80 years. There was no statistically significance difference between both groups regarding sex, age, height, weight and BMI (Table 1).

**Table 1:** **Demographic data and medical history**

	Group A	Group B	*p*value
	N: 21 patients	N: 18 patients	
Sex			0.442
Male*	7 (33.3%)	4 (22.2%)	
Female*	14 (66.7%)	14 (77.8%)	
Age (years)^	71.0 ± 5.0	70.9 ± 5.1	0.949
Weight (kg.)^	77.9 ± 5.3	77.1 ± 5.7	0.652
Height (cm.)^	174.0 ± 5.5	174.7 ± 5.5	0.687
BMI^	25.3±1.9	24.8±1.8	0.428
Primary tumor			0.480
Lung*	5 (23.9%)	6 (33.3%)	
Breast*	9 (42.9%)	9 (50%)	
Unknown*	7 (33.3%)	3 (16.6%)	
Symptoms			
Dyspnea*	20 (95.3%)	18 (100%)	0.348
Cough*	8 (38.1% )	7 (38.8%)	0.959
Chest pain*	10 (52.3%)	9 (50%)	0.882
Previous thoracocentesis			
Number/month^	4.7 ± 1.8	4.7 ± 1.5	0.926
Total amount (liters)^	2.7 ± 0.5	2.8 ± 0.4	0.246
Re-collection after (days)^	6.1 ± 2.3	6.7 ± 1.5	0.421
Relief of symptoms*	21 (100%)	18 (100%)	1.00
Complete lung inflation*	21 (100%)	16 (87.8%)	0.117

*Number (%).

^mean ± SD.

There was no statistically significance difference between both groups regarding pre-pleurodesis medical history (Table 1). Regarding patients complaints; dyspnea was present in 38 patients (97.43%), while cough was present in 15 patients (38.46%) and chest pain occurred in 19 patients (48.71%) with no statistically significance difference between both groups (Table 1). Also, there was no statistically significance difference between both groups regarding history of thoracocentesis (number of thoracocentesis per month, amount drained, number of days before recollection and relief of symptoms). The mean total pleural fluid drained ± SD was (2.7 ± 0.5 L) and (2.8 ± 0.4 L) for groups A and B, respectively with no statistically significant difference.

There was no statistically significance difference between both groups regarding physical and cytological analysis of pleural fluid (type of effusion, character and cytology) (Table 2). There was no statistically significance difference between both groups regarding biochemical analysis of pleural fluid (LDH content and total protein) (Table 2).

**Table 2:** **Physical, cytological & biochemical analysis of pleural fluid**

	Group A	Group B	*p*value
	N: 21 patients	N: 18 patients	
Type of effusion			0.493
Exudative*	4 (19.2%)	2 (11.2%)	
Transudative*	17 (80.8%)	16 (87.8%)	
Character			0.447
Hemorrhagic*	14 (66.7%)	10 (55.6%)	
Serosanguinous*	7 (33.3%)	8 (45.4%)	
Cytology			0.458
Positive malignant cells*	20 (95.3%)	16 (87.8%)	
No malignant cells*	1 (4.7% )	2 (11.2%)	
LDH content (IU/L)^	220.0 ± 95.5	296.8 ± 75.1	0.209
Total protein (g/L)^	93.1 ± 55.6	107.0 ± 59.9	0.457

*Number (%).

^mean ± SD.

IU/L: International Unit per Liter.

g/L: gram per Liter.

There was no statistically significance difference between both groups regarding post-pleurodesis success rate and response to treatment (Table 3). There was no statistically significance difference between both groups regarding post-pleurodesis complications (pain, fever, and allergy to the agent) (Table 3). The most common post-pleurodesis complication was pain (encountered in 14 patients and 9 patients in group A and group B respectively). Post-pleurodesis fever was recorded in 4 patients in each group (Table 3).

**Table 3:** **Post-procedure data**

	Group A	Group B	*p*value
	N: 21 patients	N: 18 patients	
Success rate*	17 (80.9%)	13 (72.2%)	0.519
Response to treatment			0.201
Complete inflation*	15 (71.4%)	12 (66.7%)	
Partial inflation*	2 (9.5%)	1 (5.6%)	
Failure*	4 (19%)	5 (27.8%)	
Complications			
Pain (comparative pain scale)			0.291
No pain*	7 (33.3%)	9 (50%)	
Minor pain (1-3)*	12 (57.1%)	9 (50%)	
Moderate pain (4-6)*	2 (9.5%)	0	
Severe pain (7-10)*	0	0	
Fever*	4 (19.2%)	4 (22.3%)	0.807
Allergy to agent*	2 (9.6%)	0	0.179
Post-procedure hospital stay(days)^	4.7 ± 1.2	4.2 ± 1.0	0.172
Recurrence of dyspnea*	4 (19%)	5 (27.7%)	0.519

*Number (%).

^mean ± SD.

During the long-term follow up there was recurrence of dyspnea in 4 cases with talc powder pleurodesis (19%) and in 5 cases with Povidone-iodine pleurodesis (27.8%) with no statistically significant difference between both groups.

There was no statistically significance difference between both groups regarding the post-pleurodesis hospital stay (Table 3). There was one case of mortality recorded in group A with the cause of death related to the primary tumor not the pleurodesis. No mortality was recorded in group B.

## Discussion

Recurrent and symptomatic pleural effusions are common in patients with malignancy. Up to 25% of patients with lung cancer and 50% of patients with breast cancer will develop a pleural effusion. Overall, mesothelioma, breast and lung cancer, account for the majority of malignant pleural effusions. According to underlying disease, many patients with malignant pleural effusion may live for months or even years. These patients’ quality of life is therefore of much importance and the aim of treatment should be beside the management of the primary disease, is to relieve symptoms, and to decrease the discomfort of the patient [10]. The necessity for repeated aspirations to relieve dyspnea is both physically and psychologically traumatic to the patient and a burden to the healthcare system. Therefore, the majority of patients will need a procedure to remove the fluid and prevent recurrence [11]. Treatment options for malignant pleural effusions are determined by several factors: symptoms and performance status of the patient, the primary tumor and its response to systemic therapy, and lung re-expansion following pleural fluid evacuation [12].

Pleurodesis is considered the best palliative therapy for the treatment of recurrent malignant pleural effusions [13]. Several techniques and various agents have been used for this purpose, with variable efficacy and safety [14]. Talc, tetracycline and bleomycin have been widely used for pleurodesis. Many studies have shown the effectiveness and safety of Povidone-iodine as an agent for pleurodesis with achieving very good results [3,15].

Our study included 39 cases divided into two groups; group A had talc pleurodesis and group B had povidone-iodine pleurodesis. They were 11 males (28.2%) and 28 females (77.8%) with no statistical significant difference between both groups regarding sex. Our study patients’ ages ranged from 65–80 years. Mean ± SD (71.0 ± 5.0 for group A and 70.9 ± 5.1 for group B).

Regarding patients’ complaints: the most common symptom in our study was dyspnea (100% of cases), followed by cough which occurred in 15 cases, and chest pain that occurred in 19 cases. Occurrence of dyspnea can be explained as moderate to massive pleural effusion causing compression on the lung. Also presence of cough and chest pain in some cases can be explained by the massive effusion, pleural irritation and chest infection with no statistical significant difference between both groups.

Regarding the response to treatment in group A there was complete response with no fluid re-accumulation in 15 patients (71.4%), and partial response in two patients (9.5%) with radiologically detected re-accumulation of minimal to mild amount at 2 months post procedure but never developed any clinical dyspnea during the follow-up and failure in 4 cases (19%) with recurrence of dyspnea and radiologically detected re-accumulation of moderate to massive pleural effusion. In group B, there was complete response with no fluid re-accumulation in 12 patients (66.7%), and partial response in one case (5.6%) who developed re-accumulation of fluid but never developed any clinical dyspnea, and failure in 5 cases (27.8%) with recurrence of dyspnea and radiologically detected re-accumulation of moderate to massive pleural effusion with no statistically significant difference between both groups.

In a prospective randomized study, Agarwal R. and colleagues randomly assigned patients with pleural effusion or pneumothorax, to receive chemical pleurodesis with either iodopovidone or cosmetic talc. They studied 38 patients with pleural effusions, who required pleurodesis, with the common pleural diseases being lung cancer and pulmonary tuberculosis. They observed complete success with absence of re-accumulation of fluid on CXR at 30 days in 16/19 (84.2%) in the iodopovidone group and 15/19 (78.9%) patients in the talc groups [16].

Mohsen et al. studied 44 patients with malignant pleural effusion secondary to breast cancer, divided into 2 groups using VATS talc pleurodesis in one group and bedside povidone-iodine in the other group. His study results match with our study regarding the success rate between both groups [4]. They reported no fluid re-accumulation in 19 patients (87%), and partial response in one patient (4%) and failure in two patients (9%) in the talc pleurodesis group. In the Povidone-iodine pleurodesis group, they found that there was complete response with no fluid re-accumulation in 17 patients (85%) at the early post-procedure follow-up, and failure response in three patients (15%) with no statistically significant difference between both groups which agrees with our study [4].

In a systematic review and meta-analysis on the efficacy and safety of iodopovidone pleurodesis, Agarwal R. and colleagues found that the success rate of iodopovidone pleurodesis varied from 70 to 100 per cent in different studies with the pooled success rate being 88.7 per cent (95% CI, 84.1 to 92.1) by the random effects model. The success rate was not significantly different whether tube thoracostomy or thoracoscopy was used for pleurodesis (P = 0.13) [17].

In another systematic review that included 1,168 patients, Walker-Renard PB and colleagues found that the complete success rate of talc was 93 per cent compared with Corynebacterium parvum (76%), tetracycline (67%), doxycycline (72%) and bleomycin (54%) [18].

Regarding the complications of our procedure, Chest pain and fever were the most common adverse effects in both groups. In our study, chest pain was recorded in 14 cases of group A and 9 cases of group B. Fever was the second most common complication; 4 cases in each group and anti-pyretic was given with close follow-up and fever subside with no more side effects until removal of the drain and discharge, with no statistically significant difference between both group. There was no other complication reported in our study. There was one case of mortality recorded in group A with the cause of death related to the primary tumor and not the pleurodesis. No mortality detected in group B.

In Agarwal R. and colleagues study, all patients experienced chest pain with median (IQR) Visual Analogue Scale of 20 (10–30) mm and a range of 10–90 mm. Fever occurred in nine patients (four in the iodopovidone group and five in the talc group) and was self-limited. Two patients (one in each group) developed empyema, which was treated with antibiotics. None of the patients, in their study, developed ARDS, visual loss or hypotension associated with administration of either agent [16].

Mohsen et al. agree with our results regarding post-operative complications as chest pain was the most common complications (4 cases only with talc pleurodesis) followed by fever (4 cases with talc pleurodesis, a single case with Povidone-iodine pleurodesis) but without a significant difference [4].

In their systematic review and meta-analysis, Agarwal R. and colleagues found that there were no deaths, acute respiratory distress syndrome (ARDS) or visual loss related with iodopovidone pleurodesis. They found that the complications reported of iodopovidone pleurodesis included chest pain and systemic hypotension [17].

Concerns that Povidone-iodine might be associated with visual loss were reported by Wagenfeld et al. in three cases during VATS [19]. However, authors used an unusual large amount of 200–500 ml of 10% Povidone-iodine [19]. They also noted that the safe amount to be used is 20 ml of 10% iodine, which is the amount that we have used in our study. As an additional safety precaution, we administered this dose in a diluted form (in normal saline).

The limitations of our study included the small sample size and not measuring the pH of the pleural fluid which can affect the success of pleurodesis as reported by some authors [20].

## Conclusion

Based on the results of our study, Povidone-iodine was shown to be an efficient pleurodesis agent and demonstrated a good safety profile in treating malignant pleural effusions with a good success rate and few minor complications. Therefore, it can be considered as a cost effective alternative sclerosing agent for pleurodesis when talc is not available or contraindicated.
